# Supplementary figures and images for: Aβ/Amyloid Precursor Protein-Induced Hyperexcitability and Dysregulation of Homeostatic Synaptic Plasticity in Neuron Models of Alzheimer’s Disease
Source: Front Aging Neurosci. 2022 Jul 6;14:946297. doi: 10.3389/fnagi.2022.946297 (PMC9344931; doi:10.3389/fnagi.2022.946297)

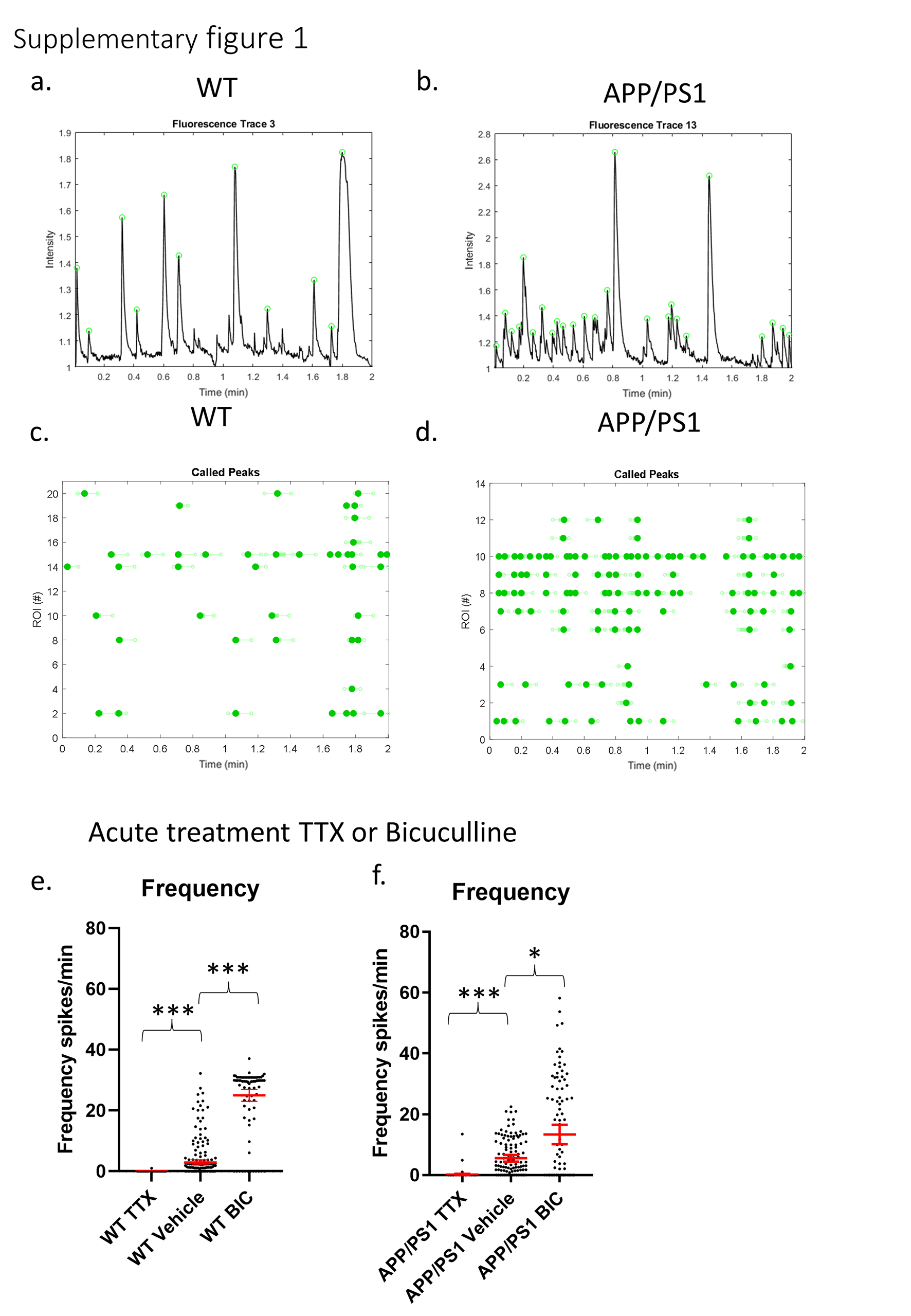

Supplement: Supplementary Figure 1 — Live-cell imaging method for assaying spontaneous calcium transients. (A,B) Representative traces of spontaneous activity over a 2 min period in neurons from WT and APP/PS1 mice, respectively. (C,D) Raster plots showing overall calcium transients (spikes represented as green dots) in WT and APP/PS1 neuron field of views (FOVs); ROI# or neuron number on the Y-axis and time in minutes on the X-axis. (E) Graph shows frequency from WT neurons treated with 1 μM TTX or 20 μM bicuculline. Note that TTX significantly reduces calcium oscillations whereas bicuculline significantly increases them compared to vehicle treated CTRL (TTX mean = 0.01429 CI = −0.01421–0.04278, n = 70; vehicle mean = 2.923, CI = 2.094–3.752, n = 209; bicuculline mean = 25.00, CI = 23.04–26.97, n = 104, p-values compared to vehicle: TTX = 0.0001 and bicuculline = 0.0001, Kruskal–Wallis test). (F) Graph demonstrating acute effects of TTX and bicuculline in APP/PS1 neurons. Note that similar to in WT neurons, TTX blocks activity while bicuculline raises activity (TTX mean = 0.1644 CI −0.07614–0.4050, n = 118; vehicle mean = 5.164, CI = 4.140–6.188, n = 188; bicuculline mean = 13.77, CI = 10.49–17.06, n = 97, p-values compared to vehicle: TTX = 0.0001 and bicuculline = 0.0379, Kruskal–Wallis test). *p < 0.05, ***p < 0.001. [file Image_1.TIF]

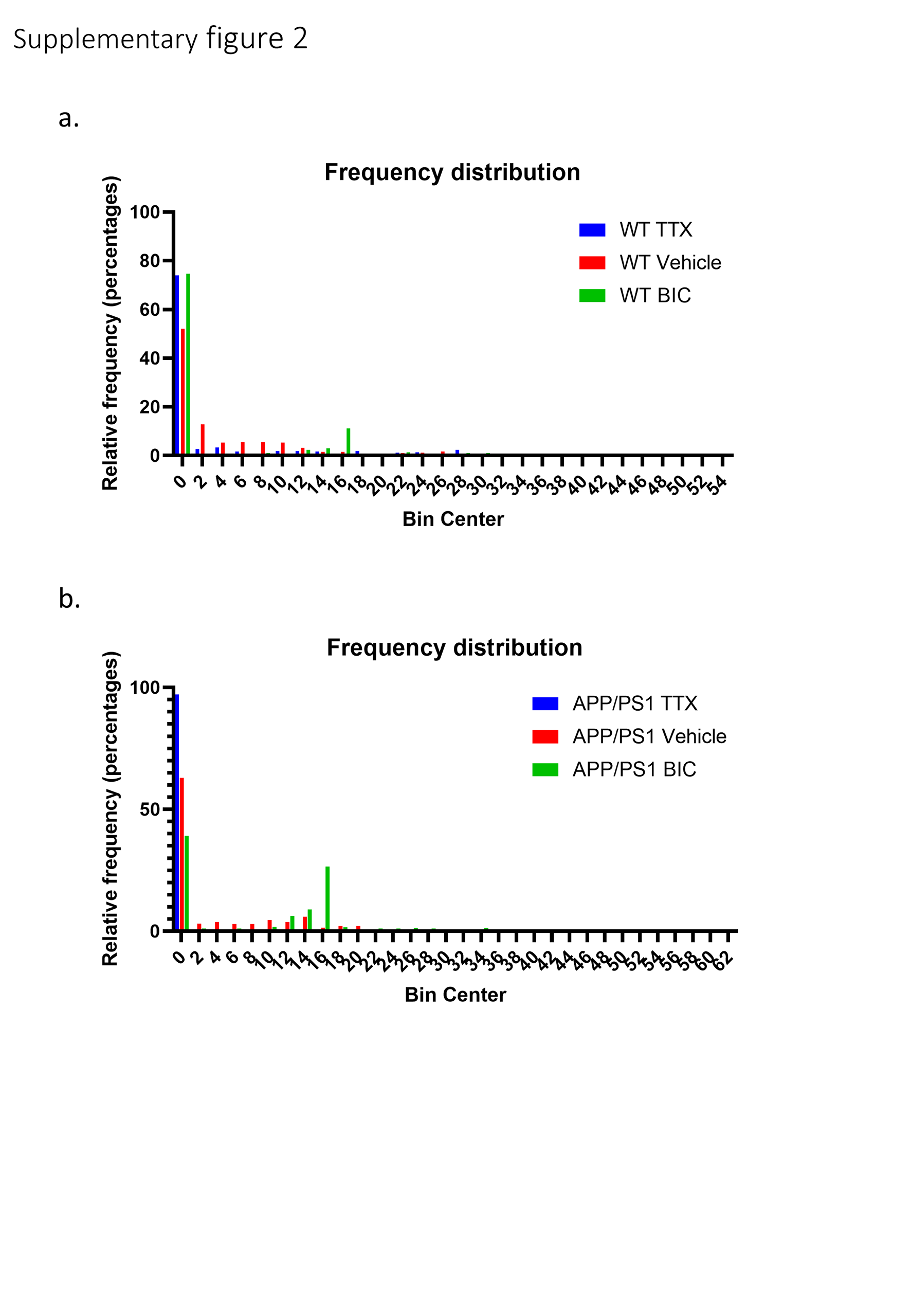

Supplement: Supplementary Figure 2 — Frequency distribution of WT and APP/PS1 after 48 h treatment with TTX or bicuculline. (A) Frequency distribution of firing frequencies of WT neurons treated with TTX, bicuculline or vehicle for 48 h; neurons shown in a graph; N = 3 cultures. (B) Frequency distribution of firing frequencies of APP/PS1 neurons treated with TTX, bicuculline or vehicle for 48 h; neurons shown in a graph; N = 3 cultures. [file Image_2.TIF]
